# **The Cytoskeletal Elements MAP2 and NF-L Show Substantial Alterations in Different Stroke Models While Elevated Serum Levels Highlight Especially MAP2 as a Sensitive Biomarker in Stroke Patients**

**DOI:** 10.1007/s12035-021-02372-3

**Published:** 2021-05-01

**Authors:** Bianca Mages, Thomas Fuhs, Susanne Aleithe, Alexandra Blietz, Constance Hobusch, Wolfgang Härtig, Stefan Schob, Martin Krueger, Dominik Michalski

**Affiliations:** 1 Institute of Anatomy, Leipzig University, Leipzig, Germany; 2 Section of Soft Matter Physics, Faculty of Physics and Geosciences, Leipzig University, Leipzig, Germany; 3 Department of Neurology, Leipzig University, Leipzig, Germany; 4 Paul Flechsig Institute of Brain Research, Leipzig University, Leipzig, Germany; 5 Department of Neuroradiology, Leipzig University, Leipzig, Germany

**Keywords:** Cerebral ischemia, Stroke, NF-L, MAP2, Biomarker, Atomic force microscopy

## Abstract

In the setting of ischemic stroke, the neurofilament subunit NF-L and the microtubule-associated protein MAP2 have proven to be exceptionally ischemia-sensitive elements of the neuronal cytoskeleton. Since alterations of the cytoskeleton have been linked to the transition from reversible to irreversible tissue damage, the present study investigates underlying time- and region-specific alterations of NF-L and MAP2 in different animal models of focal cerebral ischemia. Although NF-L is increasingly established as a clinical stroke biomarker, MAP2 serum measurements after stroke are still lacking. Therefore, the present study further compares serum levels of MAP2 with NF-L in stroke patients. In the applied animal models, MAP2-related immunofluorescence intensities were decreased in ischemic areas, whereas the abundance of NF-L degradation products accounted for an increase of NF-L-related immunofluorescence intensity. Accordingly, Western blot analyses of ischemic areas revealed decreased protein levels of both MAP2 and NF-L. The cytoskeletal alterations are further reflected at an ultrastructural level as indicated by a significant reduction of detectable neurofilaments in cortical axons of ischemia-affected areas. Moreover, atomic force microscopy measurements confirmed altered mechanical properties as indicated by a decreased elastic strength in ischemia-affected tissue. In addition to the results from the animal models, stroke patients exhibited significantly elevated serum levels of MAP2, which increased with infarct size, whereas serum levels of NF-L did not differ significantly. Thus, MAP2 appears to be a more sensitive stroke biomarker than NF-L, especially for early neuronal damage. This perspective is strengthened by the results from the animal models, showing MAP2-related alterations at earlier time points compared to NF-L. The profound ischemia-induced alterations further qualify both cytoskeletal elements as promising targets for neuroprotective therapies.

**Supplementary Information:**

The online version contains supplementary material available at 10.1007/s12035-021-02372-3.

## Introduction

As part of the neuronal cytoskeleton, neurofilaments, microtubules, and associated proteins not only maintain cellular stability but also impact on critical neuronal functions, such as the regulation of axonal caliber [1–3], conduction velocity [4], axonal transport [5–7], and synaptic function [8]. Therefore, it is not surprising that these elements play crucial roles in the pathogenesis of a variety of central nervous system disorders [9–11].

In the setting of stroke, an affection of the neuronal cytoskeleton is discussed to represent a key mechanism during the evolution of the ischemic lesion and hence the transition from reversible to irreversible tissue damage [12, 13]. Although ischemic stroke represents one of the leading causes of death worldwide [14, 15] and despite tremendous efforts in preclinical and clinical stroke research, acute therapies are still restricted to recanalizing approaches with intravenous thrombolysis and mechanical thrombectomy [16, 17]. Therefore, the need for single or adjuvant neuroprotective treatments is still evident. However, even though cytoskeletal derangements were described in the context of ischemic stroke [18–23], neurofilaments and microtubule-associated proteins have so far been largely neglected as targets for neuroprotective approaches.

As one of the four neurofilament proteins of the central nervous system, the neurofilament-light chain (NF-L) is predominantly abundant in axons, whereas smaller amounts of NF-L can also be found in dendrites and neuronal somata [23–25]. Of note, NF-L has turned into focus of stroke research, as recent clinical studies demonstrated a correlation between peripheral serum NF-L levels and the infarct volume [26–29], as well as the functional outcome and mortality of stroke patients [27, 30–35], effectively promoting NF-L as a diagnostic and prognostic biomarker.

This perspective is supported by several preclinical studies demonstrating robust affections of NF-L in diverse models of acute traumatic brain injury (TBI) and stroke as indicated by an altered NF-L-related immunofluorescence intensity in histological sections as well as decreased protein levels in the infarcted tissue [13, 20, 23, 36]. Especially the use of certain polyclonal antibodies not only detects the 68 kDa NF-L protein, but also its degradation products of smaller molecular weight allow a clear-cut detection of the ischemia-affected areas and affected neurons as indicated by an increased NF-L-related immunofluorescence intensity [20].

Another ischemia-sensitive component of the neuronal cytoskeleton is the microtubule-associated protein 2 (MAP2), which is predominantly expressed in dendrites [37–39]. Alterations of MAP2 expression and immunoreactivity were regularly observed in models of permanent focal cerebral ischemia [12, 40–42], but data on the effect of an acute vessel recanalization are missing. Furthermore, even though a preclinical stroke study could show an increase of MAP2 in the serum of animal subjected to ischemia [43], the conceivable use of MAP2 as a biomarker in the clinical setting has not yet been addressed.

To evaluate the potential of the cytoskeletal elements MAP2 and NF-L as possible neuroprotective targets, the present study was set up to investigate both proteins in a translational approach with multimodal characterizations. Immunofluorescence microscopy and Western blot analyses were correlated with ultrastructural and mechanical analyses using electron microscopy and atomic force microscopy in different animal models of stroke. In addition to models with permanent occlusion, mechanical and pharmacological recanalizing techniques were also used to mimic the clinical setting. Further, the possible use of MAP2 as a biomarker was assessed in direct comparison to NF-L in the serum of patients suffering from focal cerebral ischemia with and without recanalizing treatments.

## Material and Methods

### Study Design

This study includes preclinical and clinical data and considers modern techniques for acute vessel recanalization in both the animal experiments and the clinical investigations. First, mouse and rat brains were subjected to different types of focal cerebral ischemia in order to analyze cytoskeletal alterations using immunofluorescence microscopy, Western blotting, electron microscopy, and atomic force microscopy. Second, NF-L and MAP2 levels were measured in blood samples from patients suffering from different types of focal cerebral ischemia.

### Animal Experiments

Animal experiments included the filament-based model in 64 male C57BL/6 J mice and the thromboembolic model in 12 male Wistar rats, both leading to right-sided focal cerebral ischemia due to an occlusion of the middle cerebral artery (MCAO; details are given below). Animals were provided by Charles River Laboratories (Sulzfeld, Germany) with a mean body weight of 25 g (mice) and 290 g (rats). Both models were used to induce either permanent ischemia (pMCAO) or 60 min of transient ischemia (tMCAO) with observation periods of 4 and 24 h in mice and rats as well as 72 h in mice. While the middle cerebral artery of 4h, 24h, and 72h pMCAO animals remained occluded for the given observation times, 4h, 24h, and 72h tMCAO animals underwent either mechanical (mice) or pharmacological recanalization (rats) after an ischemic period of 60 min (details are given below). The different models are abbreviated as follows: “h” refers to the observation time, while “t/p” refers to the applied model (transient/permanent MCAO). Sufficient MCAO-related cerebral infarction was evaluated according to the standardized scoring system by Menzies et al. [44], whereas animals had to reach a minimum of 2 (decreased grip of the contralateral forelimb while tail pulled) as pre-defined study inclusion criterion.

### Ischemia Induction in Animals

MCAO surgery was performed under deep anesthesia using 2–2.5% isoflurane (Baxter, Unterschleißheim, Germany) in a mixture of 70% N_2_O/30% O_2_. In mice, MCAO was induced according to Longa et al. [45], with minor modifications as described before in detail [46]. In short, a standardized silicon-coated 6-0 filament (Doccol Corporation, Redlands, CA, USA) was inserted into the internal carotid artery and carefully moved forward to the origin of the middle cerebral artery until bending was observed or resistance felt. In rats, MCAO surgery was performed according to Zhang et al. [47], with minor modifications as described earlier [48]. In brief, a weight-adapted blood clot was prepared and later injected in the distal section of the internal carotid artery using a PE-50 catheter and a small bolus of saline. Every procedure during anesthesia was performed using a rectal probe and a thermostatically controlled warming pad adjusting the body temperature to 37°C. In order to mimic recanalizing approaches such as intravenous thrombolysis and mechanical thrombectomy as routinely applied in the clinical setting, transient MCAO (tMCAO) was accomplished by either filament removal in mice or intravenous administration of recombinant tissue plasminogen activator (rtPA, Actilyse®, Boehringer Ingelheim, Ingelheim am Rhein, Germany) in rats 60 min after ischemia induction, whereas animals with permanent ischemia (pMCAO) remained untreated.

### Fluorescence Microscopy

Animals were sacrificed and transcardially perfused with saline and 4% paraformaldehyde (PFA; Serva, Heidelberg, Germany) in phosphate-buffered saline (PBS) at pre-defined observation periods of 4, 24, and 72 h. Brains were removed from the skull and post-fixed in 4% PFA for 24 h, followed by equilibration in 30% phosphate-buffered sucrose. Forebrains were then serially cut into coronal 30-μm-thick sections with a freezing microtome (Leica SM 2000R, Leica Microsystems, Wetzlar, Germany). All brain sections were stored at 4°C in 0.1 M Tris-buffered saline, pH 7.4 (TBS), containing 0.2% sodium azide. For multiple immunofluorescence labeling, sections were blocked with 5% normal donkey serum and 0.3% Triton X-100 in TBS for 1 h and then incubated overnight with primary antibodies (Table 1) diluted in the blocking solution. The next day, sections were incubated with mixtures of appropriate secondary antibodies (Table 1) in TBS containing 2% bovine serum albumin for 1 h at room temperature. Every change of incubation medium was preluded and followed by thorough rinsing in TBS. Finally, sections were mounted onto fluorescence-free microscope slides and cover-slipped with fluorescence mounting medium (Dako North America, Inc., Carpinteria, CA, USA). Omitting primary antibodies served as control, which resulted in the absence of staining. Microscopy and image acquisition of qualitative MAP2/NF-L data were performed with the Biorevo BZ-9000 microscope (Keyence, Neu-Isenburg, Germany).

**Table 1:** Antibodies used for immunohistochemistry and Western blot

	Dilution	Manufacturer
Immunohistochemistry		
Primary antibody		
Mouse-anti-MAP2 (clone HM-2)	1:500	Sigma, Taufkirchen, Germany
Rabbit-anti-neurofilament L	1:200	Synaptic Systems, Göttingen, Germany
Secondary antibody	Dilution	Manufacturer
AlexaFluor488-donkey-anti-mouse IgG	1:250	Thermo Fisher, Waltham, MA, USA
AlexaFluor568-donkey-anti-rabbit IgG	1:250	Thermo Fisher
Western blot		
Primary antibody		
Mouse-anti-neurofilament L (clone DA2)	1:1000	Thermo Fisher
Rabbit-anti-neurofilament L	1:2000	Synaptic Systems
Mouse-anti-MAP2 (clone HM-2)	1:1000	Sigma
Mouse-anti-β-actin	1:2000	Cell Signaling, Danvers, MA, USA
Secondary antibody	Dilution	Manufacturer
HRP-horse-anti-mouse IgG	1:10000	Vector lab., Burlingame, CA, USA
HRP-goat-anti-rabbit IgG	1:10000	Vector lab.

Sample sizes used for immunofluorescence microscopy are as follows: mice, 4h-t (*n*=5), 4h-p (*n*=6), 24h-t (*n*=6), 24h-p (*n*=6), 72h-t (*n*=6), and 72h-p (*n*=2); rats, 4h-t (*n*=3), 4h-p (*n*=4); 24h-t (*n*=3), and 24h-p (*n*=2).

### Fluorescence Intensity Measurements

For quantification of fluorescence intensities, NF-L- and MAP2-labeled brain sections were scanned with an Axio Scan.Z1 slide scanner (Carl Zeiss Microscopy GmbH, Jena, Germany), and files were analyzed using the netScope Viewer Pro software (Net-Base Software GmbH, Freiburg i. Br., Germany). Two different quantifications were performed:

(a) Corresponding to data shown in Fig. 2: Images of striatal and cortical regions in mice after 4h-t, 4h-p, 24h-t, 24h-p, 72h-t, and 72h-p were captured at 10× magnification. Due to the slightly variable distribution of the ischemic lesion after MCAO [49], sections and regions of interest (ROIs) were selected based on the ischemia-induced decrease of MAP2-related immunofluorescence intensity. They were then mirrored to the contralateral hemisphere which served as control, thus capturing 4 ROIs per animal. In one 72h-p mouse brain, poor tissue integrity impeded quantification of the cortex, leading to a sample size of 72h-p: cortex, *n*=1, and striatum *n*=2, only.

(b) Corresponding to data shown in Fig. 3: To assess possible region-dependent alterations of immunofluorescence intensities, 9 ROIs were placed from non-affected medial to affected lateral regions throughout the cortex of the ischemic hemisphere of 24h-t and 24h-p mice. Thereby, ROIs 4 and 5 were strictly placed next to the ischemic border. Thus, ROIs 1–4 were always placed in non-ischemic and ROIs 5–9 in ischemic areas. On the contralateral hemisphere, 3 ROIs were mirrored to corresponding regions and served as control. To capture the immunofluorescence intensity throughout all cortical layers, each ROI had a field dimension of 200μm width and a height which was adapted to the height of the cortex at each point. Per animal and ROI, the mean fluorescence intensity of each ROI was measured using ImageJ software (National Institutes of Health, Bethesda, MD, USA) to calculate the n-fold change compared to the mean value of the 3 contralateral ROIs, which served as control.

### Western Blots

Mice were sacrificed and perfused with saline, only. Brains were removed from the skull and manually dissected. Next, stroke-affected areas, being demarked by the ischemia-associated edema, as well as the respective contralateral areas were dissected, snap-frozen in liquid nitrogen, and stored at −80°C. Each sample was homogenized and lysed by ultrasonification in 60 mM Tris-HCl, pH 6.8, containing 2% sodium dodecyl sulfate (SDS), 10% sucrose, and a protease inhibitor cocktail (Cell Signaling, Leiden, The Netherlands) on ice. Next, probes were centrifuged at 13,000 rpm and 4°C for 10 min, followed by protein concentration measurements using the BCA kit (Thermo Fisher, Waltham, MA, USA). Proteins were denatured in sample buffer (250 mM Tris-HCl, pH 6.8, containing 4% SDS, 10% glycerol, and 2% β-mercaptoethanol) at 95°C for 5 min. Then, proteins were separated using a 12.5% SDS-PAGE and transferred to nitrocellulose membranes (Th.Geyer, Renningen, Germany). Membranes were blocked with 5% dry milk in TBS (50 mM Tris-HCl, 150 mM NaCl, pH 7.5) for 30 min and incubated with primary antibodies (Table 1) at 4°C overnight. After thorough washing in buffer (6 g/l Tris, 8.8 g/l NaCl, 3 ml/l Tween 20), horseradish peroxidase–conjugated secondary antibodies (Table 1) were added for 1 h, and membranes were developed with the ECL kit (Thermo Fisher). After image acquisition, membranes were stripped with stripping buffer (15 g/l glycine, 1 g/l SDS, 10 ml/l Tween 20, pH 2.2) and reused to detect β-actin as housekeeping protein for reference. The relative protein concentration of MAP2 and NF-L was calculated from the respective β-actin-related chemiluminescence intensity. Sample sizes for Western blot analyses were 4h-t (*n*=5), 4h-p (*n*=6), 24h-t (*n*=6), and 24h-p (*n*=5).

### Electron Microscopy

Mice were sacrificed and transcardially perfused with saline, followed by perfusion with 4% PFA and 0.5% glutaraldehyde in PBS. After removal from the skull, brains were post-fixed in the same fixative for 24 h. Then, brains were cut into 60-μm-thick coronal sections using a vibratome (Leica Microsystems, Wetzlar, Germany) in cooled PBS. Sections were then transferred into PBS and stained with 0.5% osmium tetroxide (EMS, Hatfield, PA, USA) for 30 min, followed by dehydration in graded ethanol and another staining step with 1% uranyl acetate (Serva) in 70% ethanol for 1 h. Sections were further dehydrated in ethanol and propylene oxide (Sigma Aldrich, Steinheim, Germany) and then incubated in Durcupan (Sigma Aldrich). After embedding between coated microscope slides and cover slips, the Durcupan-saturated sections were polymerized at 56°C for 48 h. Areas showing stroke-associated edema were identified by light microscopy and transferred onto blocks of resin for a second polymerization step. The blocks were trimmed and cut into 55-nm-thick ultra-thin sections using an ultra-microtome (Leica Microsystems). Finally, the sections were transferred on formvar-coated grids and stained with lead citrate for 6 min. Ultrastructural analysis was performed using a Zeiss SIGMA electron microscope (Carl Zeiss Microscopy GmbH, Oberkochen, Germany).

In ischemia-affected cortical regions, the neurofilament density was analyzed in cross sections of myelinated axons. For this purpose, detectable neurofilaments were identified by diameter and morphology and counted, and the area of the cross-sectioned axon was measured to calculate the neurofilament density per μm^2^. Measurements thereby included 5 different fields of view from ischemia-affected cortical areas per animal, which were calculated as mean values and compared with contralateral unaffected regions. Of note, as a proper distinction of neurofilaments is impeded in tangentially cross-sectioned axons, the analysis was restricted to proper cross sections, while cytoplasmic areas showing organelles such as mitochondria were also excluded. Sample sizes for electron microscopy were: 4h-t and 24h-t (*n*=5, each).

### Atomic Force Microscopy

To demonstrate alterations of mechanical properties within ischemia-affected regions, atomic force microscopy (AFM) was performed in a mouse 24 h after pMCAO (24h-p). To enable the detection of ischemia-affected regions, fluorescein isothiocyanate–conjugated albumin (FITC-albumin, 0.2 mg dissolved in 0.1ml saline, Sigma, Taufkirchen, Germany) was intravenously applied to demarcate areas of ischemia-associated blood-brain barrier (BBB) breakdown 23 h after ischemia induction [50]. After a circulation time of 1h, the brain was removed from the skull and immediately cut into 350-μm-thick slices using a vibratome (HM 650V, ThermoFisher Scientific, Walldorf, Germany) in artificial cerebrospinal fluid (ACSF, containing 2.5 mM KCl, 260 mM D-Glucose, 26 mM NaHCO_3_, 1.25 mM NaH_2_PO_4_, 2 mM Na-pyruvate, 3 mM myo-inositol, 0.4 mM ascorbic acid, 1 mM MgCl_2_, 2 mM CaCl_2_, 20 mM Hepes, pH 7.4). Subsequently, slices were glued (Histroacryl, Braun, Melsungen, Germany) onto microscope slides followed by measurement of the elastic strength (Young’s modulus) in the ischemia-affected and contralateral cortex. The AFM used is a NanoWizard 4 with 300μm HybridStage (JPK, Berlin, Germany) combined with an Axio Zoom.V16 (Zeiss, Oberkochen, Germany) for fluorescence imaging [51]. The AFM image and optical image are recorded in a common reference system using the AFM software calibration routine. A CONT (Nanoworld, Neuchâtel, Switzerland) contact mode cantilever (spring constant 213mN/m) was modified with a 6-μm-diameter polystyrene bead to increase contact area. Force ramps were recorded with the following parameters: maximum force 7.5 nN, z-speed 20 μm/s, z-length 30 μm, 2048 Hz capture rate, and 10 μm data point spacing (Suppl. Figure 1 A & B). AFM data was first analyzed with the JPK data processing software to calculate the Young’s modulus using a Hertz fit to the smoothed and baseline corrected force-indentation curves (Suppl. Figure 1C). Data was post-processed with a custom written Matlab program (MathWorks, Natick, MA, USA) to calculate local averages and construct the overlay of AFM data with the whole-slice overview shown in Fig. 6. In the ischemia-affected cortex, the elastic strength of the tissue was recorded over a distance of approximately 1100 μm starting from the area of evident FITC-albumin extravasation across the border zone of detectable FITC-albumin extravasation, which delineates the infarcted tissue [52–54]. Mean values of 11 positions were calculated, with each position measuring 100×100 μm and consisting of 100 single measurements. In the contralateral cortex, 3 different positions (total 300 data points) served as control. During the whole procedure, the coronal brain slices were kept submerged in ACSF to prevent dehydration. Sample size is 24h-p (*n*=1).

**Fig. 1:**
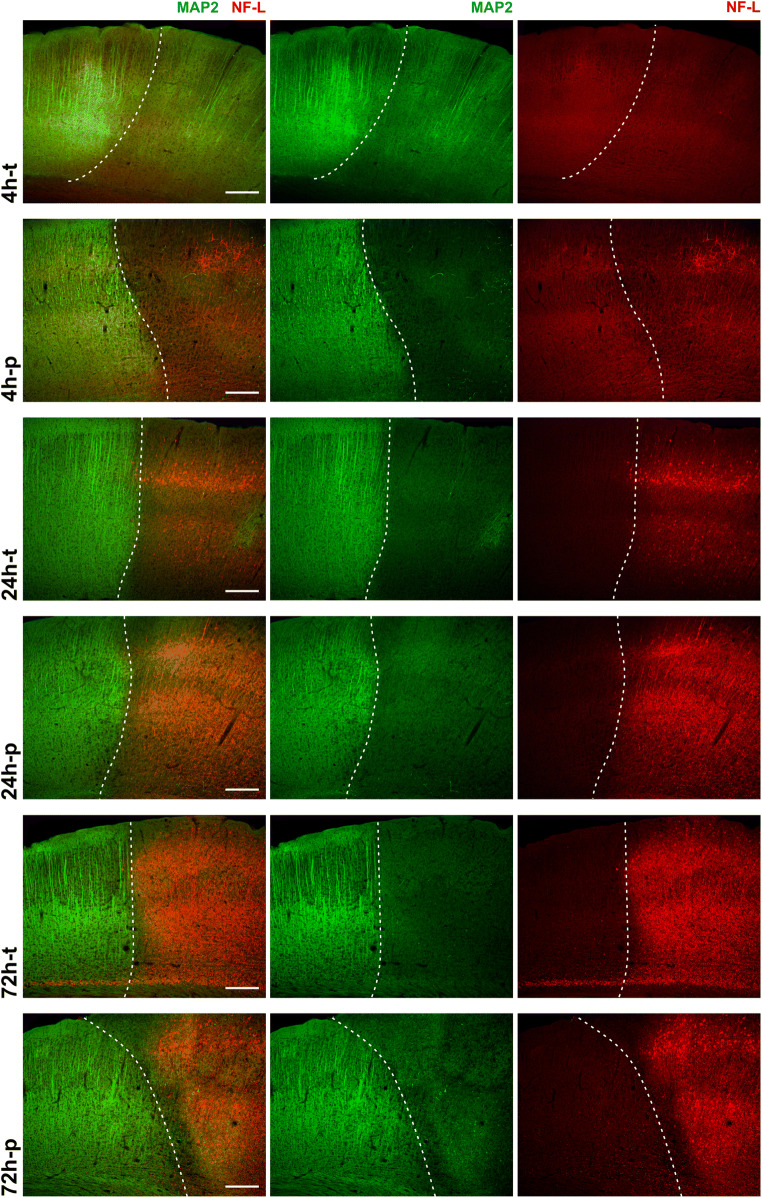
Immunofluorescence labeling of MAP2 and NF-L at different time points in the mouse model of MCAO: the reduction of MAP2-related immunoreactivity (green) identifies the infarct border (dashed line) in the cortex of mice at all time points of transient and permanent ischemia. The NF-L-related immunoreactivity (red) is increased in the infarct area, with a clear increase after 24h and 72h, but no apparent increase after 4h-t. Scale bars: 200μm

### Blood Sampling in Patients Suffering from Focal Cerebral Ischemia

In a prospective, non-interventional study, a total of 81 patients, hospitalized at the stroke unit of the Department of Neurology, Leipzig University, were included. Blood samples were collected at two pre-defined time points: 12–24 h (“day 1”) and 3–5 days (“day 3”) after hospital admission. Main inclusion criteria were (a) ischemic stroke, defined by a sudden onset of a focal neurological deficit independent of the vascular territory with evidence of a cerebral infarction either on computed tomography or magnetic resonance tomography, and (b) transient ischemic attack (TIA) with a naturally lacking evidence for a cerebral infarction on radiological examination [55]. Main exclusion criteria were any kind of known other cerebral pathologies such as neurodegenerative or inflammatory disorders as well as intracerebral hemorrhage. Patients were categorized for (a) “TIA,” (b) “stroke,” and (c) “stroke with intervention,” which represent patients who underwent a therapy aiming to re-establish cerebral blood flow (i.e., intravenous thrombolysis and/or mechanical thrombectomy). Furthermore, imaging data were used to categorize for (a) “lacunar infarcts” (diameter ≤1.5 cm) and (b) “larger infarcts” (diameter >1.5 cm). For descriptive analyses, additional data were recorded concerning the severity of the neurological deficit (National Institute of Health Stroke Scale, NIHSS) at hospital admission as well as the individual infarct etiology and medical history.

### Enzyme-Linked Immunosorbent Assay (ELISA)

Human blood samples were allowed to coagulate for 1 h at room temperature and then centrifuged at 3500 × g for 10 min. Thereafter, the supernatant was aliquoted and stored at −80°C until further processing. Serum protein levels were measured using commercial ELISA kits from Abbexa (Cambridge Science Park, Cambridge, United Kingdom; NF-L, Cat.# abx258398; MAP2, Cat.# abx358608). Analyses were performed according to the instructions given by the manufacturer. The optical density (OD) was measured at 450nm using a Mithras LB940 microplate reader (Berthold Technologies, Bad Wildbad, Germany), and then protein concentrations were calculated by extrapolation of the linear portion of the standard curve. The following sample sizes were achieved: TIA, MAP2 (*n*=12) and NF-L (*n*=12); stroke, MAP2 (*n*=26) and NF-L (*n*=27); stroke with intervention, MAP2 (*n*=21) and NF-L (*n*=20); lacunar infarct, MAP2 (*n*=17) and NF-L (*n*=16); and larger infarct, MAP2 (*n*=29) and NF-L (*n*=30).

### Statistical Analyses

Data analysis was performed using Graph Pad Prism 5.01v (GraphPad Software Inc., La Jolla, CA, USA) and the SPSS software package version 25 (IBM SPSS Statistics for Windows, IBM Corp., Armonk, NY, USA). The Grubbs’ or ROUT test was used to check for statistical outliers, and the Kolmogorov-Smirnov test (SigmaStat; v3.10, San Jose, CA, USA) was used to check for normal distribution of the data. After confirmation of a normal distribution, ANOVA followed by Bonferroni’s multiple comparison post hoc test was used to check for statistically significant differences between three or more groups. Non-normally distributed data of two dependent groups were analyzed with the Wilcoxon signed-rank test, while two independent groups were analyzed using the non-parametric Mann-Whitney U test. Non-normally distributed data of three or more independent groups were analyzed using the Kruskal-Wallis test followed by Dunn’s multiple comparison post hoc test. For analysis of the human serum samples, the potentially confounding factors age and hypertension were ruled out by additional calculations (not shown). In general, with the significance level α=0.05, *p*<0.05, indicated statistically significant differences.

## Results

### MAP2-Related Immunofluorescence Intensity Shows Early and Long-Lasting Reductions, While NF-L-Related Immunofluorescence Intensity Is Increased Throughout Later Time Points

Although MAP2 and NF-L have particularly proven as ischemia-sensitive elements of the neuronal cytoskeleton in various studies [13, 20, 23, 36, 40, 41, 56], a simultaneous region- and time-dependent characterization among different models of focal cerebral ischemia is not yet available. Therefore, immunofluorescence labeling of NF-L and MAP2 was performed throughout the applied models and time points. Of note, ischemia-associated alterations are comprehensively indicated by changes of NF-L- and MAP2-related immunofluorescence intensity throughout the applied models in mice (Fig. 1). While the ischemic region is regularly characterized by a decreased MAP2-related immunofluorescence intensity, the NF-L-related immunofluorescence intensity appears to be increased 24 and 72 h after ischemia induction. However, only a discontinuous reduction of the MAP2-related immunofluorescence intensity is observed after 4 h of transient ischemia with no apparent increase of the NF-L-related immunofluorescence intensity in the ischemic region. The latter becomes increasingly detectable after 4 h of permanent ischemia and later time points, which is then accompanied with a widespread and continuous reduction of the MAP2-related immunofluorescence intensity (Fig. 1).

Similar alterations of immunoreactivity were observed for the translationally relevant thromboembolic model of MCAO in rats 4 and 24 h after permanent and transient ischemia, the latter following pharmacological recanalization (Suppl. Figure 2). In each model, the MAP2-related immunofluorescence intensity was diminished in the ischemic region, whereas the NF-L-related immunofluorescence intensity was increased in the same area.

**Fig. 2:**
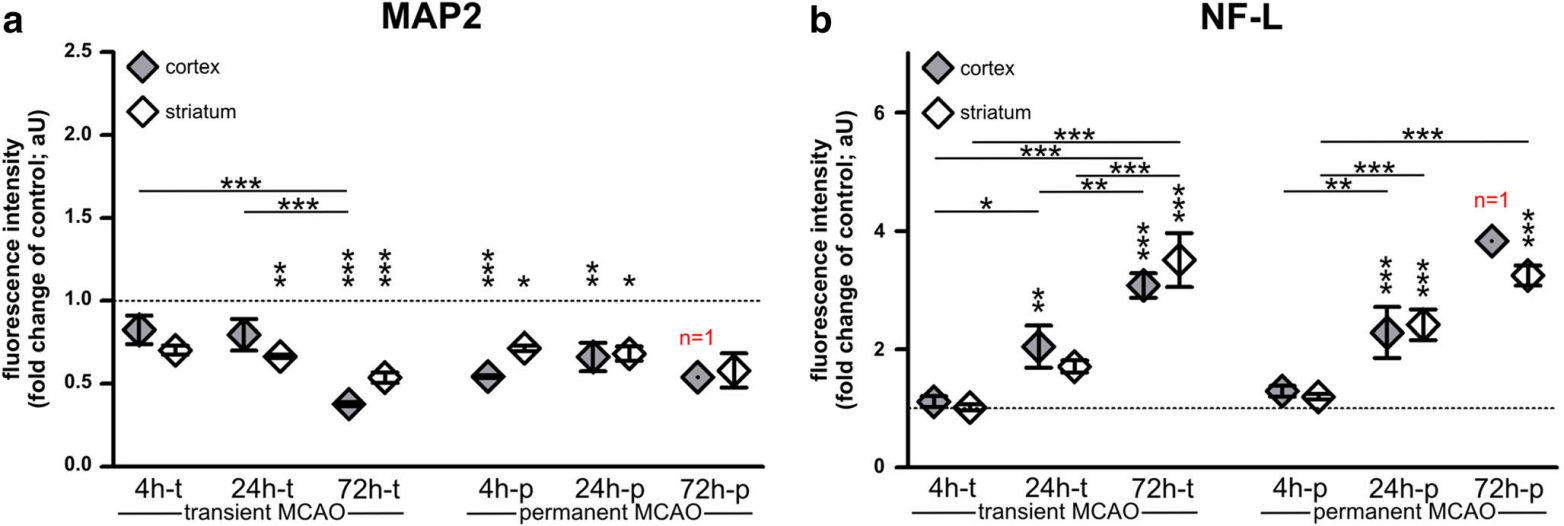
**a** The fluorescence intensity of MAP2 is regularly reduced, including early time points, but did not reach statistical significance after 4h of transient ischemia. **b** NF-L-related fluorescence intensities in the infarct area are significantly and progressively increased, but not yet after 4h of transient or permanent ischemia. Sample sizes: each group, *n*=5–6; except for 72h-p: *n*=1–2. Data are presented as mean values; error bars indicate the standard error. *p<0.05, **p<0.01, ***p<0.001

By quantification of the MAP2- and NF-L-related immunofluorescence intensities (Fig. 2) in the applied mouse models, the reduction of MAP2-related immunofluorescence intensity in stroke tissue was found to be significant after 4h-p, 24h-t, 24h-p, and 72h-t compared to control regions. In addition, the MAP2-related immunofluorescence intensity appeared to be significantly lower after 72h-t compared to 4h-t and 24h-t. Numerically, the MAP2-related immunofluorescence intensity of 72h-p infarct tissue decreased just as much (72h-p cortex, 0.541 [arbitrary unit, aU]; 72h-p striatum, 0.581 [aU]) but represented sample sizes of *n*=1 (cortex) and *n*=2 (striatum), only. Consequentially, cortical 72h-p data was not considered for statistical analysis.

The increase of the NF-L-related immunofluorescence intensity was not yet significant after 4h-t and 4h-p but increased progressively at later time points. Hence, NF-L-related immunofluorescence intensity was significantly higher in ischemic areas compared to the contralateral hemisphere after 24h-t, 24h-p, 72h-t, and 72h-p. The increase of the NF-L-related immunofluorescence intensity from early to late time points even proved to be significantly different between each time point, with the exception of 24h-p to 72h-p. Interestingly, neither the MAP2- nor the NF-L-related immunofluorescence intensity differed significantly between transient and permanent ischemia (4h-t vs. 4h-p; 24h-t vs. 24h-p; 72h-t vs. 72h-p) (Fig. 2).

### Alterations of MAP2-Related Immunofluorescence Intensity Are Uniformly Apparent Throughout the Ischemic Area, While Alterations of NF-L-Related Immunofluorescence Intensity Are Regionally Different

**Fig. 3:**
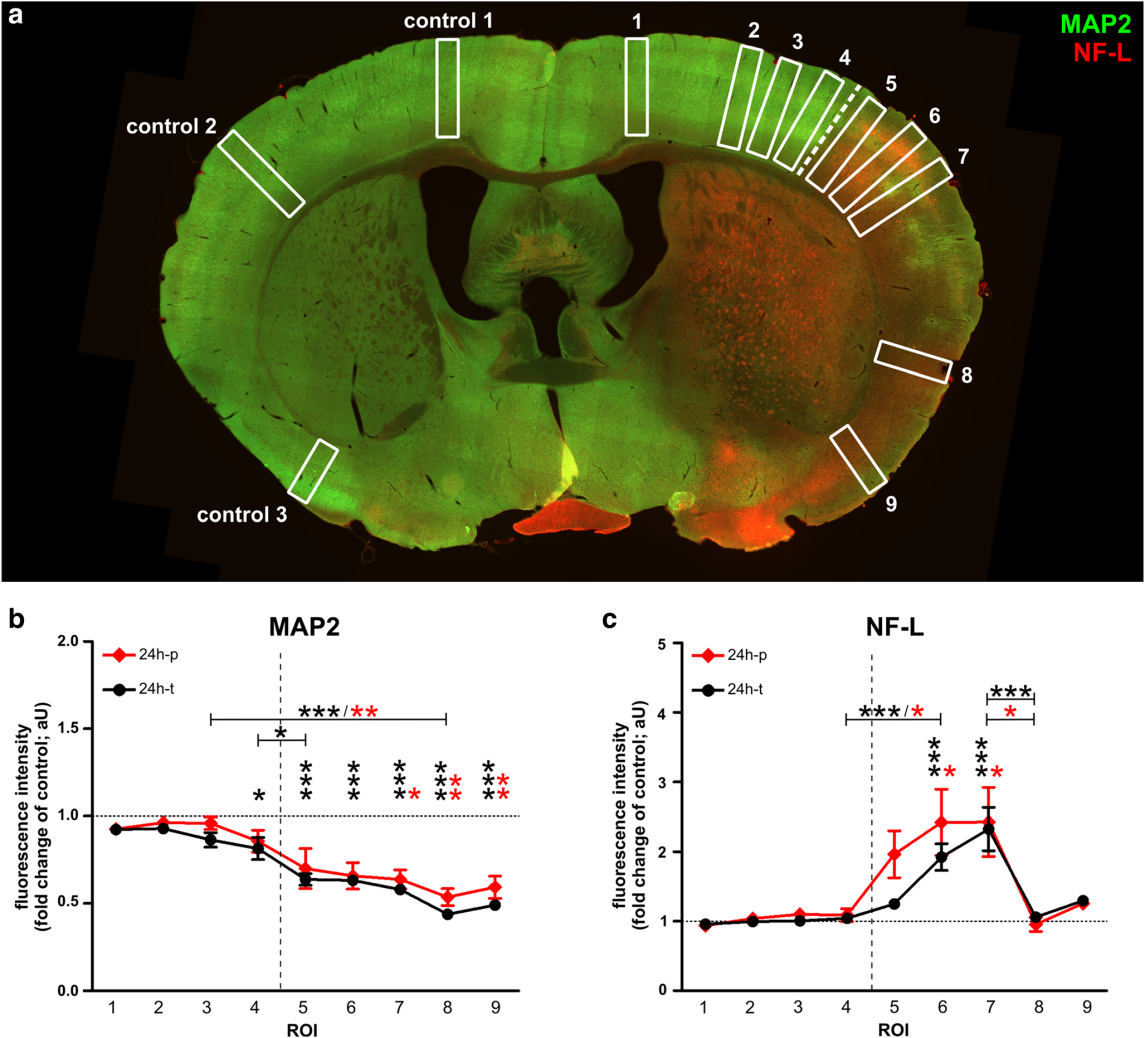
**a** The illustration indicates regions of interest (ROIs) used for quantifications of MAP2- and NF-L-related immunofluorescence intensities after 24h of transient and permanent MCAO in mice. **b** When compared to the contralateral hemisphere, the MAP2-related immunofluorescence intensity was significantly reduced throughout the infarcted cortex, **c** whereas the increase of NF-L-related immunoreactivity was only significant in ROIs of the somatosensory cortex. Neither MAP2- nor NF-L-related immunofluorescence intensities differed significantly between transient and permanent MCAO. The dashed line represents the infarct border. Sample sizes in **b** and **c**: *n*=6 for all ROIs. Data are presented as mean values; error bars indicate the standard error. *p<0.05, **p<0.01, ***p<0.001

After showing time-dependent alterations of NF-L and MAP2, further analyses addressed possible region- or reperfusion-specific differences. Therefore, multiple ROIs were placed throughout the cortex of 24h-t and 24h-p mice (Fig. 3a). Here, fluorescence intensity measurements (Fig. 3b and c) showed an abrupt and significant decrease of the MAP2-related immunofluorescence intensity at the infarct border (ROI 4 to ROI 5; 24h-t), thereby coinciding with an increase of the NF-L fluorescence intensity which became significant at ROIs 6 and 7 (24h-t and 24h-p). Furthermore, the MAP2-related immunofluorescence intensity was significantly altered in each ROI within the ischemic area, whereas alterations of NF-L-related immunofluorescence intensity were only significant in the somatosensory cortex (ROIs 6 and 7). Of note, a detrimental effect of the recanalizing approach was not observed as neither MAP2- nor NF-L-related immunofluorescence intensities differed significantly when comparing transient and permanent ischemia (24h-t and 24h-p).

### Decrease of MAP2 Protein and NF-L Protein Degradation in Stroke Tissue

**Fig. 4:**
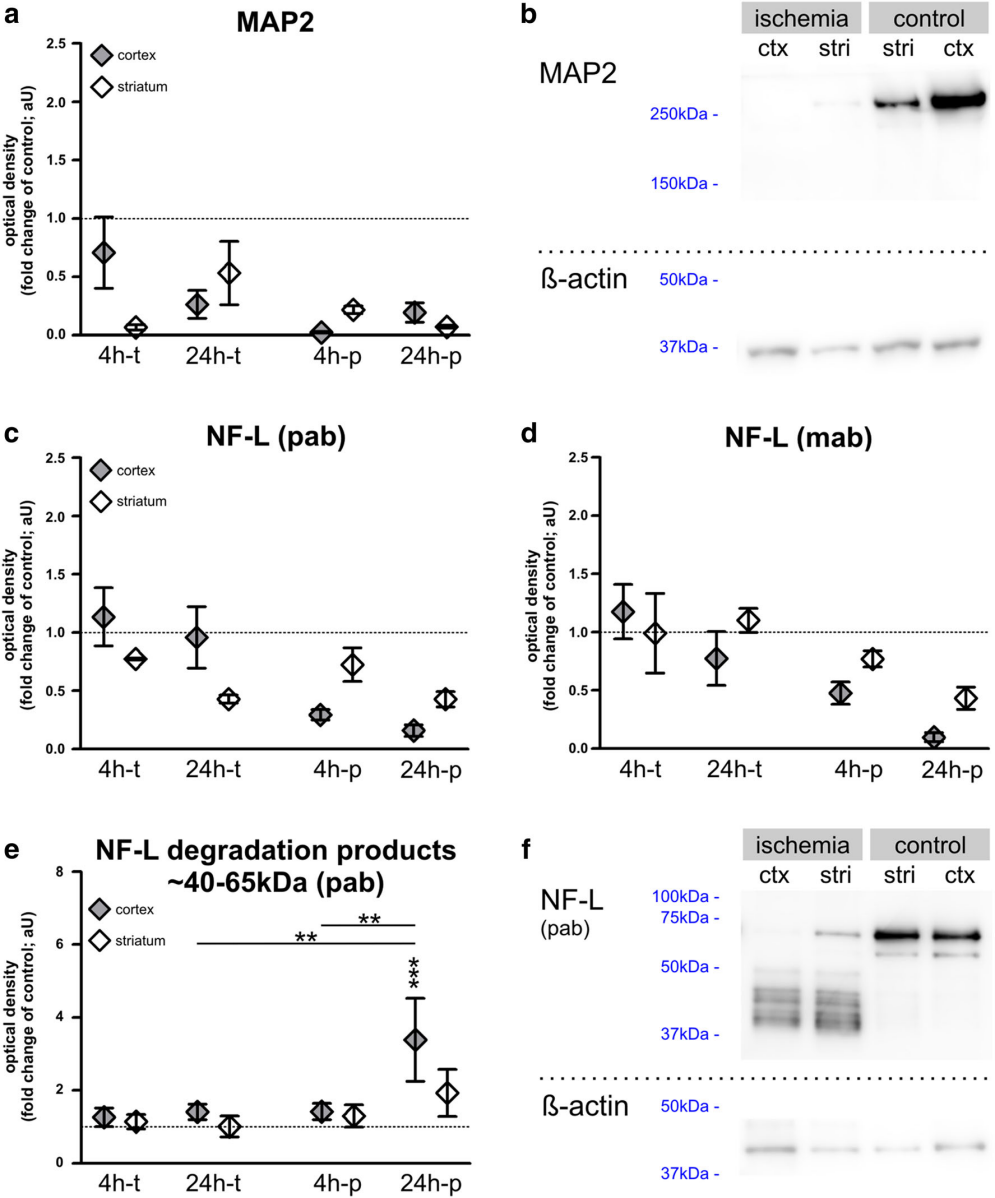
**a**, **b** Western blot analyses of MAP2 and NF-L in mice after 4 and 24 h of transient and permanent MCAO: MAP2 levels decrease in stroke tissue throughout the applied models, although without reaching significance. **c–f**) Western blot was performed with a polyclonal “pab” NF-L antibody (**c**, **e**, **f**) and a monoclonal antibody “mab” (**d**). Thereby a decrease of NF-L in infarcted tissue was measured, which was most pronounced after 24h-p, but failed to reach statistical significance. NF-L levels after 4h-t remained mostly unaltered. **e**, **f** NF-L degradation products of 40-65 kDa are significantly increased after 24h-p. *n*=5–6. Data are presented as mean values; error bars indicate the standard error. **p<0.01, ***p<0.001

Next, we performed Western blot analyses using 4h-t, 4h-p, 24h-t, and 24h-p mouse tissue to investigate whether the abovementioned alterations are reflected by changes of the MAP2 and NF-L protein levels in ischemic brain tissue. Western blots were also used to determine the abundance of NF-L degradation products, which are held responsible for the increase of NF-L-related immunoreactivity in ischemic brain tissue [20]. Importantly, in line with the decreased MAP2-related immunofluorescence intensity observed in histological sections, Western blot analyses confirm decreased MAP2 protein levels in ischemia-affected tissue for each investigated time point (Fig. 4a and b), although not reaching statistical significance.

Using two different NF-L antibodies, a trend toward reduced NF-L protein levels was observed after 4 and 24 h of permanent ischemia (Fig. 4c and d), while the transient model only exhibited a comparable reduction of NF-L protein levels in striatal areas of 24h-t animals using the polyclonal antibody, however, failing statistical significance. Of note, selective analysis of NF-L degradation products (~40-65 kDa) revealed a significant increase in the ischemic cortex after 24 h of permanent ischemia (Fig. 4e and f). Here, a 3.38-fold increase of NF-L degradation products was measured compared to controls, which also reached significance compared to cortical MCAO tissue of 4h-p and 24h-t animals.

### Ultrastructural Analysis Confirms Cytoskeletal Alterations in Ischemia-Affected Areas

**Fig. 5:**
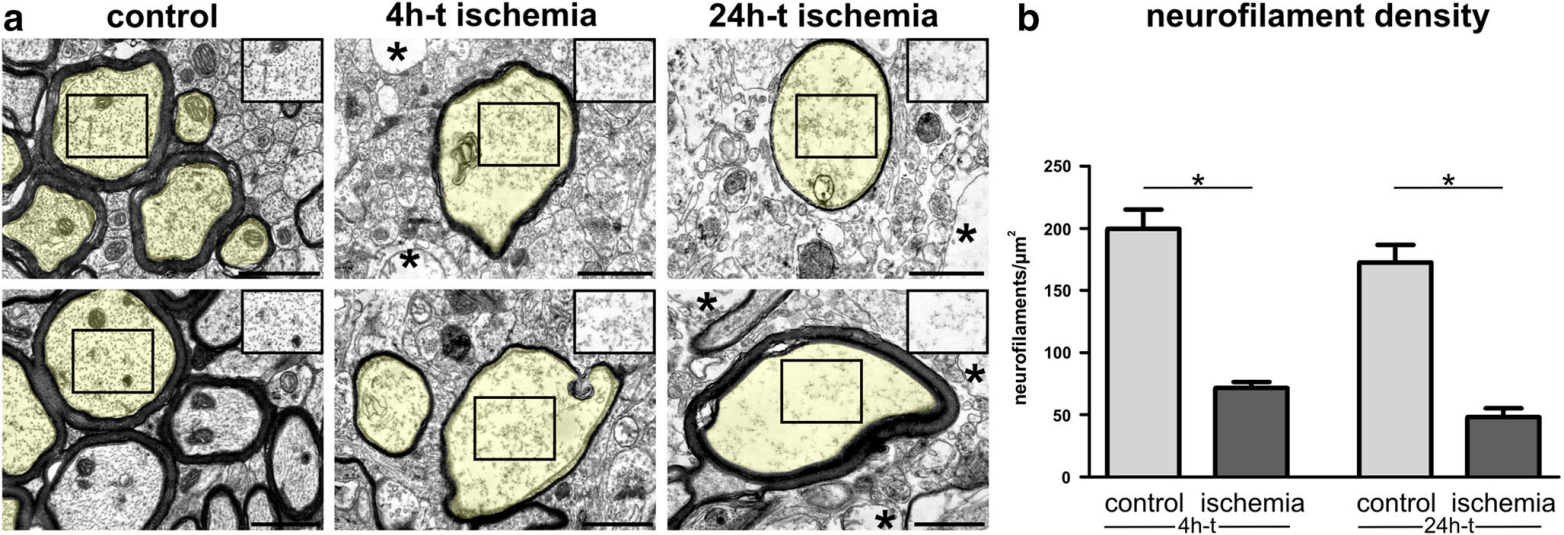
**a** Electron microscopy images show cross sections of cortical axons after 4h and 24h of transient ischemia in mice and unaffected contralateral regions. For illustration, transversally sectioned axons are transparently highlighted in yellow. The regular distribution of axonal neurofilaments is lost (insets) 4h and 24h after tMCAO. An ischemia-induced cellular edema is indicated by a less electron dense cytoplasm in the highlighted axons and insets as well as in non-axonal structures (asterisks). Please note that the images and insets also contain other components of the axonal cytoplasm which were not considered for the analysis of neurofilament density. **b** The density of detectable neurofilaments is decreased in ischemia-affected cortical axons 4h and 24h after tMCAO. *n*=5. Data are presented as mean values; error bars indicate the standard error. *p<0.05. Scale bars: 1 μm

To investigate whether the described alterations of cytoskeletal proteins are also reflected at an ultrastructural level, we applied electron microscopy of ischemia-affected cortical regions and contralateral non-affected areas of 4h-t and 24h-t animals. To allow a proper differentiation of neurofilaments from other components of the axonal cytoplasm, the analysis was restricted to axons showing strictly transversally sectioned neurofilaments, only. Here, the detectable neurofilaments were counted to calculate the neurofilament density per μm^2^. Of note, axons in ischemia-affected cortical areas regularly appeared less electron dense, which is indicative of an ischemia-associated cellular edema (Fig. 5a). Here, the homogeneous distribution of neurofilaments is lost throughout the cytoplasm (Fig. 5a). Further, the density of detectable neurofilaments was significantly reduced compared to control areas in both 4h-t and 24h-t animals (Fig. 5b).

### Mechanical Tissue Properties Are Significantly Altered in Ischemia-Affected Tissue

**Fig. 6:**
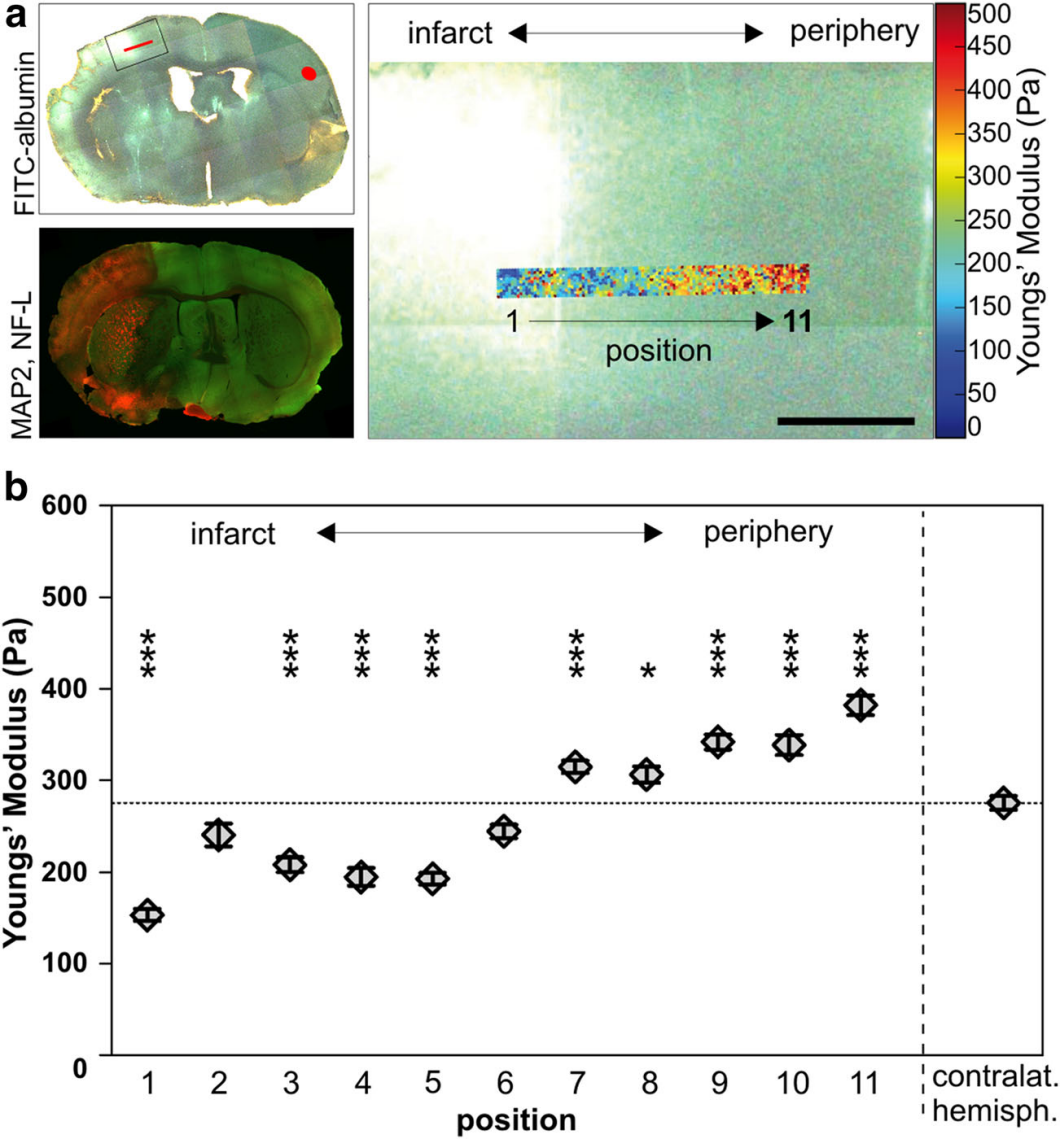
The elastic modulus of infarcted brain tissue, an indicator of tissue elasticity, was measured in a cortex of a 24h-p mouse by AFM indentation. **a** Upper left image: Overview of the measured brain slice illustrates the localization of the measurements. The ischemia-affected tissue was identified by areas of FITC-albumin extravasation (green/white). To enhance the visibility of the contralateral hemisphere on behalf of figure generation, the image was acquired with fluorescence and transmitted light. Lower left image: Overview of MAP2- (green) and NF-L (red)-labeled coronal brain slice captured with epifluorescence microscopy to illustrate that the AFM measurements comply with the infarct border visualized by immunofluorescence labeling. Right image: Higher magnification of the inset (upper left image) showing an overlay of the infarct border and the heat map (blue corresponds to softer and red to stiffer regions) of the AFM measurements. **b** Over a span of 1100μm (positions 1–11), measurements revealed decreased values of tissue elasticity in central regions (1, 3, 4, 5), whereas peripheral regions (7, 8, 9, 10, 11) exhibited an elevated elastic modulus. Each region includes 100 individual measurements. Data are presented as mean values; error bars indicate the standard error. Scale bar: 500 μm

As the cytoskeleton is presumed to ensure cellular integrity and mechanical stability, we tried to determine whether the described alterations of neuronal cytoskeletal proteins are reflected by altered mechanical properties of the ischemic tissue. Therefore, acute brain slices of a 24h-p mouse brain were prepared in order to measure the elastic strength (Young’s modulus) in ischemia-affected cortical areas and contralateral regions using atomic force microscopy. Therefore, ischemia-affected areas were identified by FITC-albumin extravasations indicative of an ischemia-associated BBB breakdown. Here, areas near to the center of the observed FITC-albumin extravasation showed a significantly decreased elastic strength compared to contralateral regions (positions 1, 3, 4, and 5 in Fig. 6a and b). In contrast, peripheral border zones not showing FITC-albumin extravasation exhibited significantly increased values (positions 7–11). Of note, the measured regions correspond to regions of the previously described MAP2 and NF-L alterations.

### MAP2 Serum Concentrations Are Significantly Increased in Stroke Patients and Appear to Be More Sensitive to Ischemia than NF-L

In order to determine whether the tissue-specific alterations observed in the animal models correspond to elevated serum concentrations of MAP2 and NF-L in human stroke patients, blood samples of 81 patients (Table 2) were analyzed at day 1 and day 3 after ischemia onset as indicated by the awareness of neurological symptoms. At hospital admission, patients suffering from TIA are naturally less affected when compared to patients suffering from a stroke with or without intervention, as demonstrated by the NIHSS.

**Table 2:** Patients’ characteristics

	Overall sample (*n*=81)	TIA (*n*=17)	Stroke (*n*=37)	Stroke with intervention (*n*=27)
Basic data				
Age (M ± SD)	66.5 ± 13.9	66.4 ± 15.8	69.0 ± 13.0	63.2 ± 13.6
Gender (female *n* (%))	31 (38.3)	10 (58.8)	15 (40.5)	6 (22.2)
Clinical data				
NIHSS (M ± SD) at admission	4.4 ± 6.1	0.5 ± 0.8	2.2 ± 2.0	9.9 ± 7.7
Infarct size				
Lacunar, diameter ≤1.5cm (*n* (%))	24 (29.6)	-	17 (45.9)	7 (25.9)
Large, diameter >1.5cm (*n* (%))	40 (49.4)	-	20 (54.1)	20 (74.1)
None (*n* (%))	17 (21.0)	17 (100)	-	-
Infarct location				
Middle cerebral artery (*n* (%))	35 (43.2)	-	18 (48.7)	17 (63.0)
Anterior cerebral artery (*n* (%))	2 (2.5)	-	2 (5.4)	-
Posterior cerebral artery (*n* (%))	8 (9.9)	-	7 (18.9)	1 (3.7)
Vertebrobasilar territory (*n* (%))	10 (12.3)	-	6 (16.2)	4 (14.8)
Multiple locations (*n* (%))	9 (11.1)	-	4 (10.8)	5 (18.5)
None (*n* (%))	17 (21.0)	17 (100)	-	-
Infarct etiology				
Large artery atherosclerosis (*n* (%))	18 (22.2)	1 (5.9)	10 (27.0)	7 (26.0)
Artery to artery embolism^$^ (*n* (%))	31 (38.3)	7 (41.1)	16 (43.3)	8 (29.6)
Cardioembolism^§^ (*n* (%))	15 (18.5)	2 (11.8)	5 (13.5)	8 (29.6)
Small vessel occlusion (*n* (%))	4 (4.9)	1 (5.9)	3 (8.1)	-
Others (*n* (%))	5 (6.2)	2 (11.8)	1 (2.7)	2 (7.4)
Unknown (*n* (%))	8 (9.9)	4 (23.5)	2 (5.4)	2 (7.4)
Medical history				
Arterial hypertension (*n* (%))	67 (82.7)	12 (70.6)	33 (91.9)	21 (77.8)
Diabetes mellitus (*n* (%))	32 (39.5)	4 (23.5)	21 (56.8)	7 (25.9)
Nicotine^#^ (*n* (%))	39 (48.1)	7 (41.2)	19 (51.4)	13 (48.1)
Hyperlipidemia (*n* (%))	50 (61.7)	9 (52.9)	22 (59.5)	19 (70.4)
Atrial fibrillation (*n* (%))	11 (13.6)	1 (5.9)	3 (8.1)	7 (25.9)
Treatment				
Intravenous thrombolysis (*n* (%))		-	-	14 (51.9)
Endovascular treatment (*n* (%))		-	-	7 (25.9)
Combined approach (*n* (%))		-	-	6 (22.2)
Time point of blood collection				
Onset to 1 collection (days M ± SD)	1.1 (0.6)	1.1 (0.7)	1.3 (0.7)	0.9 (0.3)
Onset to 2 collection (days M ± SD)	3.5 (0.8)	3.4 (0.7)	3.7 (0.9)	3.2 (0.8)

^#^Including current and previous nicotine consumption

^$^Including relevant carotid macroangiopathy and carotid plaque load based on ultrasound evaluation without consecutive stenosis of less than 50% but with suspected artery to artery embolism

^§^Etiology was also rated as cardioembolism in the absence of atrial fibrillation in cases with relevant cardiac pathology, i.e., persistent foramen ovale

**Fig. 7:**
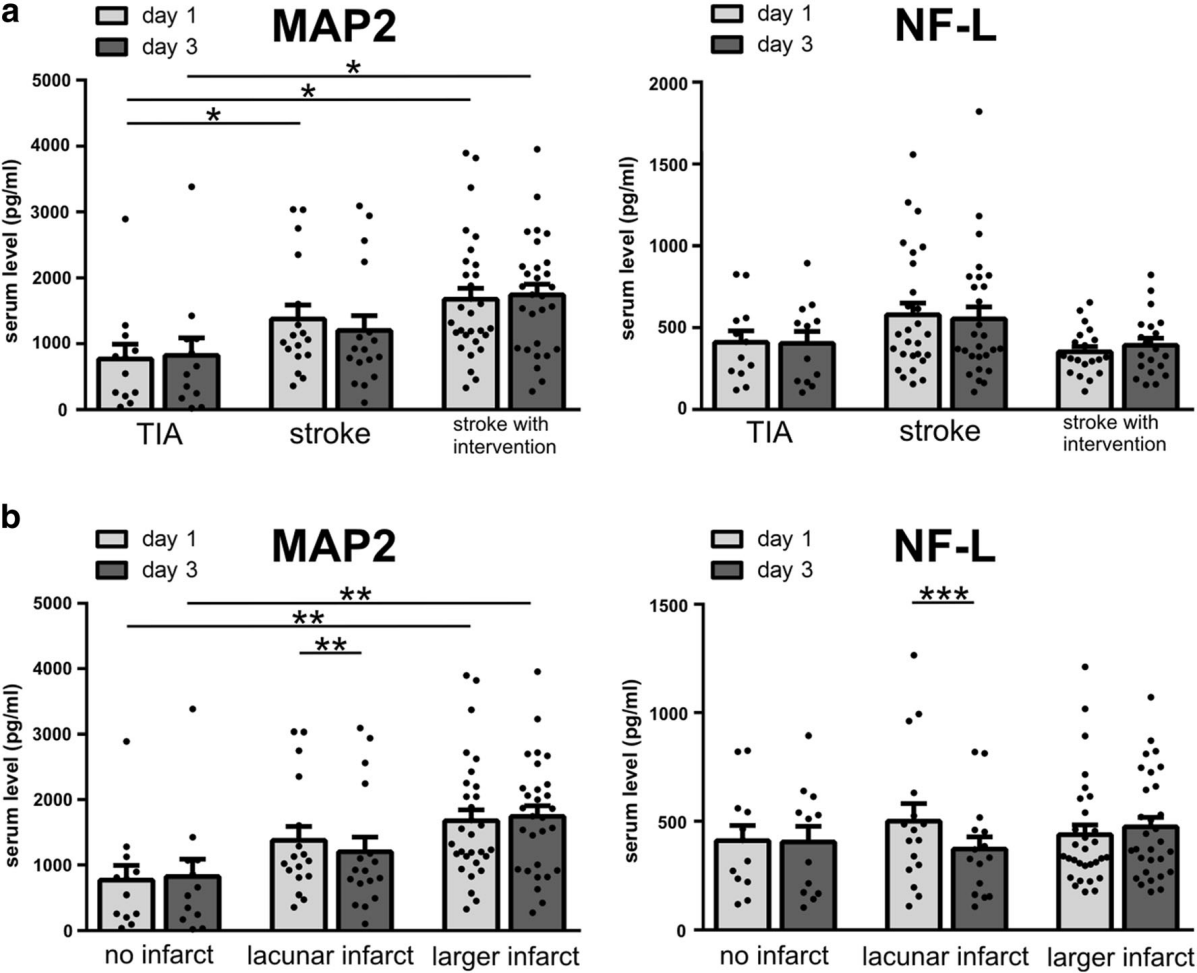
MAP2 and NF-L serum levels of patients suffering from stroke at day 1 and day 3 after stroke onset: patients were divided into groups depending on **a** stroke intervention or **b** the size of the ischemic lesion. Patients with stroke (with and without intervention) have significantly higher serum concentrations of MAP2 than patients suffering from TIA. Significantly higher MAP2 serum levels are also found in patients suffering from larger infarcts compared to no ischemic lesion. Significant differences between day 1 and day 3 are only found after a lacunar stroke (MAP2 and NF-L). **a** and **b**: *n*=12–30. Bars represent mean values; dots represent individual values; error bars indicate the standard error. *p<0.05, **p<0.01, ***p<0.001

Accordingly, significantly increased MAP2 serum levels were observed when comparing TIA patients with patients suffering from stroke, with or without intervention (Fig. 7a). Moreover, patients exhibiting larger infarct sizes showed significantly elevated MAP2 serum levels, although a stepwise increase in patients with lacunar lesions was not observed (Fig. 7b). NF-L levels were found to increase numerically in patients suffering from stroke, but did not differ significantly from TIA patients (Fig. 7a). Although the increase of NF-L serum levels was not found to be statistically significant, the levels numerically decreased after recanalization to levels comparable to TIA patients, while this post-interventional reduction was not observed for MAP2 levels. Further, a significant reduction of MAP2 and NF-L serum levels over time was observed in patients with lacunar stroke (Fig. 7b).

Similar to the described results, the comparison between TIA patients and a combined group including patients with and without intervention (Suppl. Figure 3) also exhibited significantly different levels of MAP2 between TIA and stroke patients at day 1 (*p* = 0.0012) and day 3 (*p* = 0.0079) with higher values in stroke patients, while serum levels of NF-L failed to provide a significant difference (day 1 *p* = 0.5531; day 3 *p* = 0.4457).

As significant elevations of blood serum levels are not observed for NF-L, MAP2 is likely to represent a more sensitive biomarker for ischemia-induced tissue damage. However, neither NF-L nor MAP2 serum levels showed a significant correlation to the severity of the neurological deficit as assessed by the NIHSS (not shown).

## Discussion

This study explores alterations of the cytoskeletal elements NF-L and MAP2 in the setting of stroke in order to evaluate their potential use for neuroprotective approaches and biomarker analyses. Considering evident translational hurdles from bench to bedside [57–59], this study for the first time addresses cytoskeletal elements in a combined preclinical and clinical setup with comparable timelines and interventions regarding recanalizing techniques. To allow a spatio-temporal characterization of cytoskeletal alterations, animal experiments were furthermore performed with different observation periods after ischemia induction.

In general, neurofilaments and MAP2 have been recognized as elements of the neuronal cytoskeleton, which are exceptionally prone to ischemia-mediated damage [18–20, 22, 23, 40]. Therefore, both proteins have turned into focus in stroke research as alterations of the neuronal cytoskeleton have been considered to mediate the transition of a reversible to an irreversible neuronal damage and to maintain cellular integrity [12, 13, 25]. Moreover, their clinical use as biomarkers correlating with the severity of neural tissue damage has been repeatedly suggested [28, 31, 35, 43, 60, 61]. Especially the neurofilament subunit NF-L is increasingly acknowledged as biomarker in neurological disorders. Upon axonal damage and neuronal damage or death, NF-L is first released into the interstitial fluids and then to the cerebrospinal fluid and to the blood where its increased levels can be detected [26, 27, 32, 62, 63]. On the other hand, despite its acute sensitivity to ischemic insults [36, 40, 64] and its ischemia-associated degradation by the Ca^2+^-dependent proteases calpain and caspase [38, 39], the value of MAP2 as a potential biomarker in the clinical setting of stroke has so far been largely neglected.

### Alterations of MAP2 and NF-L in the Applied Animal Models of Cerebral Ischemia

In line with reports describing early ischemia-associated loss of MAP2 [39, 40, 65], the here presented immunofluorescence and Western blot analyses in mice and rats revealed that an ischemia-associated reduction of MAP2 occurs early (4h after MCAO) and uniformly throughout the infarct area (Figs. 1, 2, and 3, Suppl. Figure 2). An exception represented the model of 4 h transient MCAO, where the demarcation of the infarct border was not yet pronounced (Fig. 1). Interestingly, both Western blot and immunofluorescence analyses revealed an abrupt, but constant reduction of MAP2 levels in 4h-p tissue, which did not further decrease over time. This observation may be explained by the fact that the expansion of the infarct already reaches its maximum within 3 h in the model of permanent MCAO and further highlights the rapid and prolonged loss of MAP2 due to ischemia [66].

Contrary to the reduced immunofluorescence intensity of MAP2 in infarcted regions, the NF-L-related immunofluorescence intensity is apparently increased in ischemia-affected areas. This contradictory finding may be explained by the calpain- and caspase-dependent degradation of neurofilaments [13, 38, 39]. In line with previous reports [20, 67, 68], the therefore increased availability of smaller protein fragments of lower molecular weight can also be detected by available polyclonal antibodies used for immunofluorescence labeling (Fig. 4e and f). Consequentially, the increased immunoreactivity of NF-L in ischemia-affected areas has to be interpreted with caution, as it does not reflect an overall increase of NF-L protein levels but likely originates from the abundance of NF-L protein fragments [20].

### MAP2 as a Biomarker for Early Neuronal Damage After Stroke

Even though MAP2 serum levels were shown to increase in an animal model of experimental cerebral ischemia [43] and in patients suffering from TBI [61], clinical data of stroke patients are still lacking. The possible use of MAP2 as a stroke biomarker was therefore addressed in comparison to NF-L, which is already conceived as a valuable biomarker for acute neuronal damage [26, 27, 31].

Our preclinical data suggests a high sensitivity of MAP2 to ischemia, which is also reflected by our analyses of human blood samples, as patients suffering from stroke exhibited significantly higher MAP2 serum levels than patients suffering from TIA (Fig. 6a). Furthermore, the MAP2 serum analyses indicate a correlation with the size of the ischemic lesion, showing significantly higher values in patients with larger ischemic lesions compared to patients with small ischemic lesions.

Of note, these clinical observations are supported by our preclinical immunofluorescence experiments, showing a uniform reduction of MAP2 is throughout the lesion, whereas NF-L exhibits regional differences. As MAP2 was further shown to be reduced earlier due to ischemia than NF-L (Figs. 1, 2, 4), these regional differences may reflect the heterogeneous distribution of NF-L [69] or also regional differences of the ischemic affection within the ischemic lesion [70, 71]. Therefore, compared to MAP2, serum levels of NF-L are likely to have higher fluctuations between individuals in dependence of the ischemic region and thus a lower correlation with the infarct size. Although NF-L levels were shown to correlate with the infarct volume or the severity of clinical symptoms as assessed by the NIHSS [28, 29, 32–34], methodological or regional differences may explain why these correlations could not be confirmed in this study.

There has further been evidence that an acute loss of MAP2 may not necessarily imply neuronal death but that a MAP2 recovery might be possible after an injury of moderate severity such as after moderate TBI [65]. This is of special interest, as MAP2 blood levels in rats subjected to MCAO were shown to peak even before the appearance of ischemic lesions could be visualized by triphenyl tetrazolium chloride (TTC) staining [43]. Since the lesion volume identified by TTC staining correlates with volume of the lesion as identified by magnetic resonance or computed tomography imaging [72–75], MAP2 blood measurements might potentially capture early neuronal damage that is not yet detectable with standard neuroimaging techniques.

Taking these aspects into consideration and given that the serum levels of MAP2 exhibited a more pronounced increase compared to NF-L, MAP2 is likely to represent a more sensitive biomarker for early ischemia-induced neuronal damage.

### Reperfusion Versus Permanent Occlusion

With reference to a postulated reperfusion injury [76], the present study also included models of permanent and transient ischemia in mice and rats at different time points. Here, immunofluorescence and Western blot analyses of MAP2 and full-protein NF-L did not reveal significant differences between transient and permanent models (Figs. 1, 2, 3, and 4). Thus, neither the dreaded reperfusion injury [76] nor any benefits of reperfusion [77] could be captured by analyzing MAP2 or NF-L in the applied animal models.

Of note, although the analysis of human serum levels only exhibited a numerical trend toward decreased NF-L serum concentrations in stroke patients who underwent recanalizing therapies, the resulting values after acute therapy are comparable with TIA patients and therefore comply with studies suggesting serum NF-L measurements as a useful tool to monitor treatment responses in neurological diseases [78, 79].

Interestingly, the level of MAP2 serum concentrations appeared to be higher in stroke patients with therapies aiming on vessel recanalization than in stroke patients without such treatments. This observation is in excellent accordance with previous experiments in rats, where the amount of MAP2 released into the blood was markedly increased by reperfusion [43]. A long-lasting release of MAP2 into the blood is also described in survivors of severe TBI, where MAP2 levels were significantly higher in patients with high cognitive function compared to patients in a vegetative state [61]. Considering the dynamic functions of MAP2 in the growth, differentiation, and plasticity of neurons, especially in dendrites [80], the presented results suggest that elevated MAP2 serum levels might serve as a biomarker not only for acute neuronal damage but also for neuronal regeneration after stroke. Since remodeling and restored dendrite function can be observed in peri-infarct neurons after stroke [81], MAP2 serum level alterations might well represent the fraction of moderately, potentially reversibly damaged neurons of the penumbra.

### Ultrastructural Alterations and Impaired Tissue Integrity

To investigate whether the described ischemia-induced alterations of the neuronal cytoskeleton can also be captured at an ultrastructural level, electron microscopy was applied to analyze the neurofilament density of cortical axons (Fig. 5). The here observed decrease of neurofilament density appears in line with the above-described reduction of NF-L protein as well as of other neurofilament proteins due to ischemia [20]. Of course, the here captured neurofilaments only represent the proportion of neurofilaments, which remained detectable by electron microscopy in the analyzed ischemia-affected axons, while degradation products or smaller protein fragments may not remain distinguishable from other ischemia-affected components of the axonal cytoplasm. These degraded protein fragments [67, 68], which are likely responsible for the observed increase of NF-L-related immunofluorescence intensity, cannot be recognized with this method. Therefore, the reduced density of detectable neurofilaments cannot represent a direct measure of the protein concentration. Nevertheless, the described ultrastructural alterations can be correlated with the observed reduction of NF-L and MAP2 protein levels (Fig. 4) as well as their altered patterns of immunofluorescence intensity.

Further, the confirmation of a cellular edema in the analyzed regions is in line with the reported cytotoxic edema of cells in the ischemia-affected areas including the ischemic penumbra [82, 83] and thereby also correlates with an impaired but potentially restorable neuronal function [70, 84]. However, in the analyzed axons, the reduced electron density of the axonal cytoplasm is likely to represent both an increased water content due to a cellular edema and an impaired cytoskeletal network, as shown by the reduced neurofilament density.

As an impaired function of the neuronal cytoskeleton also impacts on cellular integrity [13], we further explored mechanical properties of the ischemia-affected tissue with atomic force microscopy. Importantly, the elastic strength was found to be decreased in central regions of the ischemic tissue, whereas increased values were found in peripheral regions (Fig. 6). These supposedly contradictory findings illustrate the naturally expected variations of ischemia-induced tissue affection with different degrees of tissue damage according to the penumbra concept [70, 82, 84]. The varying mechanical properties are likely to be explained by more pronounced tissue damage in the center of the ischemic lesion, where irreversible tissue damage leads to loss of cellular integrity and thus decreased values of the elastic strength. Since MAP2 is an important regulator of the neuronal cytoskeleton which stabilizes microtubules, bundles actin filaments, and cross-links the cellular cytoskeleton [85, 86], the reduction of MAP2 levels in the ischemic region is likely to explain the described softening of the tissue as a consequence of an impaired cytoskeleton.

Further, intermediate filaments such as neurofilaments are known to enable cells to withstand severe deformations [87]. Vice versa, the ultrastructural confirmation of decreased neurofilament densities could also be linked to the decreased tissue stiffness.

In contrast, in peripheral areas of the ischemic penumbra, the intracellular edema in combination with preserved cellular integrity could be responsible for the increased elastic strength in these regions.

Other possible reasons for the observed stiffening could relate to increased actin (stress) fiber generation as reaction to oxygen depletion [88, 89] or to reactions caused by changes of local pH [90] in areas of potentially reversible tissue damage. Although AFM measurements can be influenced by various variables, the presented values of the elastic strength appear to be plausible since comparable values were observed when analyzing living neurons under cell culture conditions [91]. Since AFM measurements could not directly be performed on the MAP2-/NF-L-immunolabeled sections for technical reasons, identification of the infarcted tissue was ensured by detection of areas showing FITC-albumin extravasation. Similar to areas of increased NF-L immunoreactivity, FITC-albumin extravasation was shown to include regions of the ischemic penumbra [20, 54] and therefore areas of potentially salvageable tissue [70].

### Summary and Conclusion

In a translational approach with different animal models as well as human serum samples, the present study reveals an early and uniform reduction of MAP2 throughout the ischemic lesion. Furthermore, a regionally dependent increase of NF-L-related immunofluorescence intensity is demonstrated in the ischemic tissue, which most likely originates from the binding of NF-L degradation products. The ischemia-induced reduction of MAP2 and NF-L full-length protein is further confirmed at the protein level, at the ultrastructural level, and by a decreased elastic strength of the ischemia-affected tissue. Since the described alterations are shown to include areas of the penumbra [20, 54], NF-L and MAP2 might represent promising targets for upcoming neuroprotective therapies. Importantly, a direct comparison of NF-L and MAP2 in human blood samples highlights MAP2 as the more sensitive biomarker in stroke patients.

## Supplementary information

Supplementary Figure 1A) Schematic of the used AFM setup: The sample is positioned in a Petri dish and submerged in ACSF. The AFM scan head approaches the sample from above. An upright long working distance fluorescent microscope sits above the AFM scan head. This allows fluorescence and AFM imaging in a common reference frame without moving the sample. B) AFM measurement principle: Elasticity is measured by recording force-indentation curves on the sample. The cantilever, a tiny leaf spring, approaches the sample vertically from above. After making contact it is pushed into the sample until a given force setpoint is reached. The cantilever is then retracted and the next point is measured. C) AFM force indentation curve: The contact position is defined as zero position for the vertical axis with positive values indenting into the tissue. The part of the curve above the sample is used to define the zero force baseline. The part indenting into the tissue is fitted with the Hertz model for contact between a sphere and a half-space, $F=\frac{4}{3}E{R}^{\frac{1}{2}}{d}^{\frac{3}{2}}$, where F is force, E the Young’s Modulus, R the indenter radius, and D the indentation. (PNG 544 kb)

High resolution (TIF 15624 kb)

Supplementary Figure 2 MAP2 and NF-L immunofluorescence labeling of rats subjected to 4 or 24 h of transient or permanent MCAO: a pronounced loss of MAP2-related immunoreactivity (green) identifies the cortical infarct border (dashed line) at each time point. NF-L-related immunoreactivity (red) is increased in the infarct area, with the most pronounced increase after 24h-p. Scale bars: 100 μm (PNG 3826 kb)

High resolution (TIF 6093 kb)

Supplementary Figure 3 MAP2 and NF-L serum levels of patients suffering from stroke at day 1 and day 3 after stroke-onset: TIA patients are compared to stroke patients (combined group, includes patients with and without intervention). Serum samples of stroke patients exhibit significantly higher serum concentrations of MAP2 than patients suffering from TIA. Bars represent mean values; dots represent individual values; error bars indicate the standard error. TIA: n=12, stroke: n=46-47. **p<0.01. (PNG 103 kb)

High resolution (TIF 1901 kb)

## Data Availability

The datasets analyzed during the current study are available from the corresponding author on reasonable request.

## Code Availability

Matlab code used in AFM data processing is available from TF upon reasonable request.
