# Supplementary figures and images for: The Cytoskeletal Elements MAP2 and NF-L Show Substantial Alterations in Different Stroke Models While Elevated Serum Levels Highlight Especially MAP2 as a Sensitive Biomarker in Stroke Patients
Source: Mol Neurobiol. 2021 May 1;58(8):4051–69. doi: 10.1007/s12035-021-02372-3 (PMC8280005; doi:10.1007/s12035-021-02372-3)

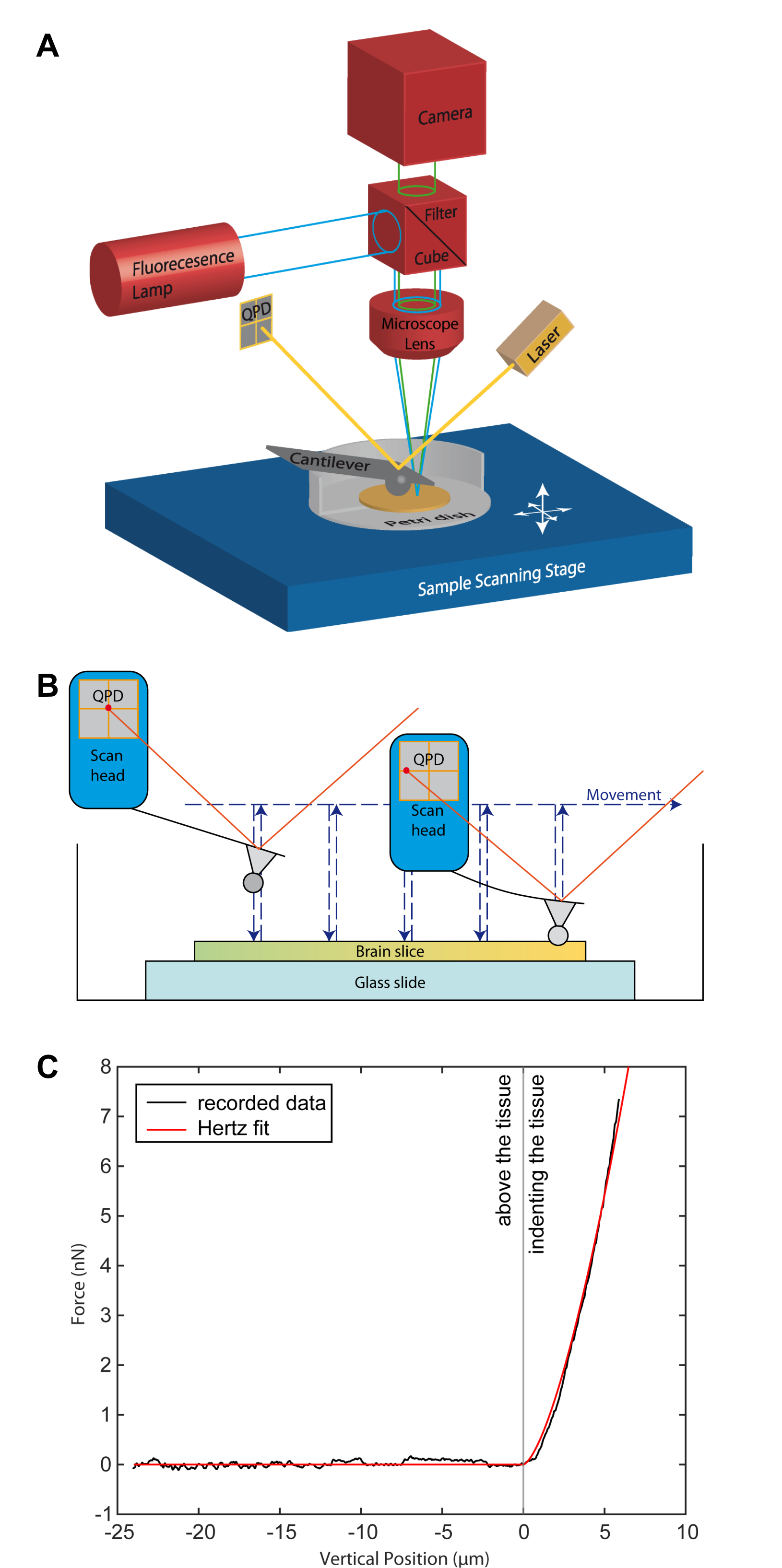

Supplement: Supplementary file 1 — A) Schematic of the used AFM setup: The sample is positioned in a Petri dish and submerged in ACSF. The AFM scan head approaches the sample from above. An upright long working distance fluorescent microscope sits above the AFM scan head. This allows fluorescence and AFM imaging in a common reference frame without moving the sample. B) AFM measurement principle: Elasticity is measured by recording force-indentation curves on the sample. The cantilever, a tiny leaf spring, approaches the sample vertically from above. After making contact it is pushed into the sample until a given force setpoint is reached. The cantilever is then retracted and the next point is measured. C) AFM force indentation curve: The contact position is defined as zero position for the vertical axis with positive values indenting into the tissue. The part of the curve above the sample is used to define the zero force baseline. The part indenting into the tissue is fitted with the Hertz model for contact between a sphere and a half-space, $F=\frac{4}{3}E{R}^{\frac{1}{2}}{d}^{\frac{3}{2}}$F=43ER12d32, where F is force, E the Young’s Modulus, R the indenter radius, and D the indentation. (PNG 544 kb) [file 12035_2021_2372_Fig8_ESM.png]

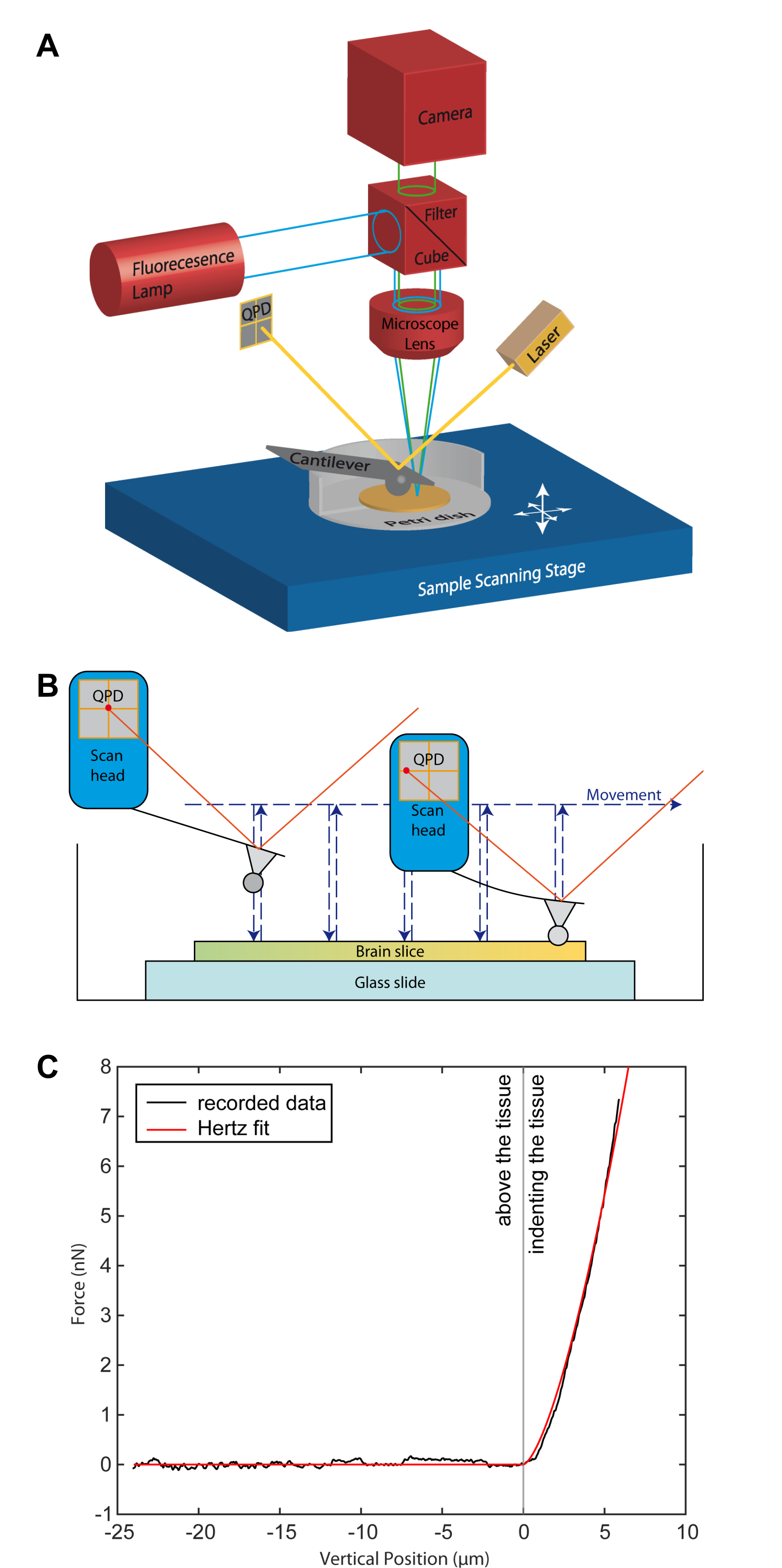

Supplement: Supplementary file 2 — High resolution (TIF 15624 kb) [file 12035_2021_2372_MOESM1_ESM.tif]

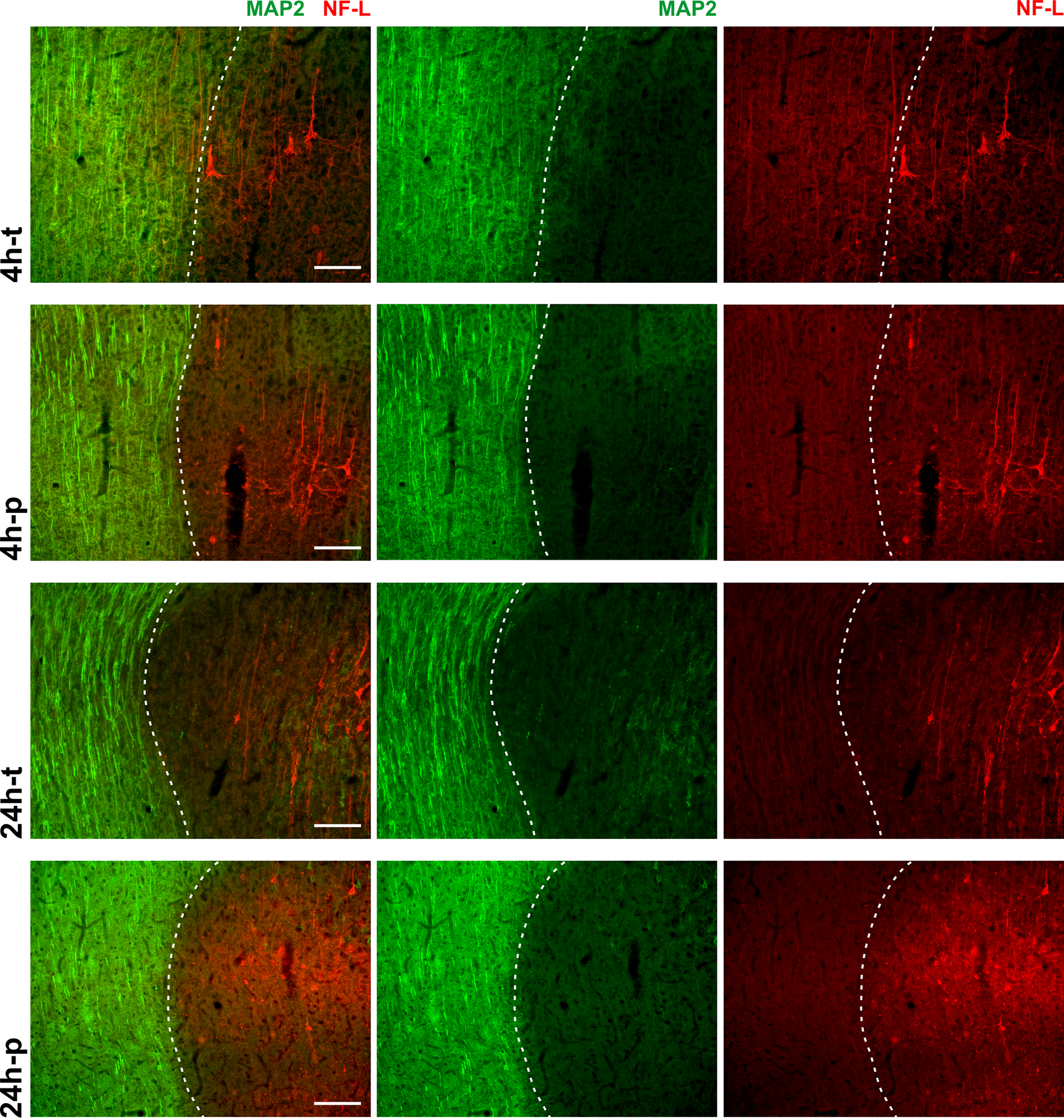

Supplement: Supplementary file 3 — MAP2 and NF-L immunofluorescence labeling of rats subjected to 4 or 24 h of transient or permanent MCAO: a pronounced loss of MAP2-related immunoreactivity (green) identifies the cortical infarct border (dashed line) at each time point. NF-L-related immunoreactivity (red) is increased in the infarct area, with the most pronounced increase after 24h-p. Scale bars: 100 μm (PNG 3826 kb) [file 12035_2021_2372_Fig9_ESM.png]

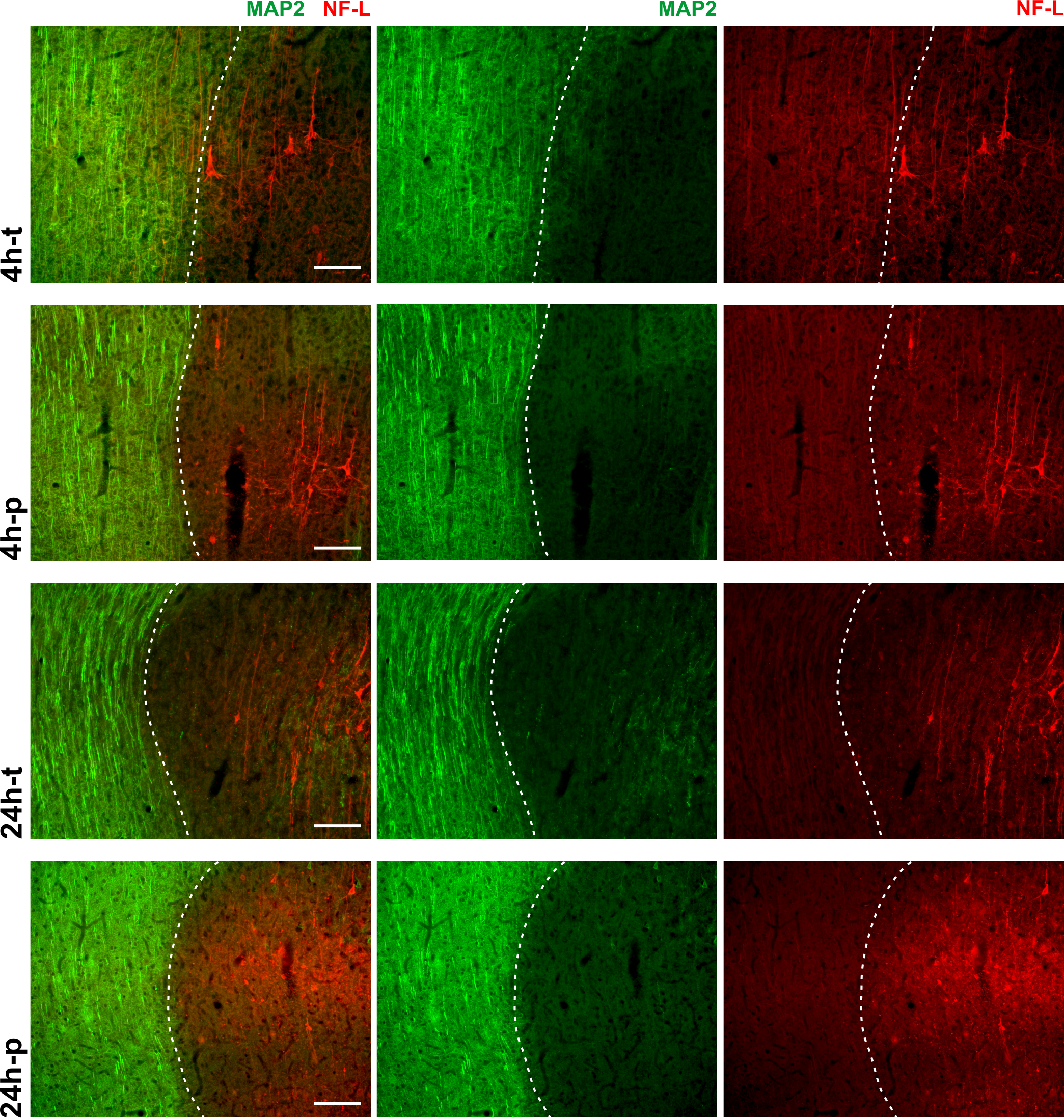

Supplement: Supplementary file 4 — High resolution (TIF 6093 kb) [file 12035_2021_2372_MOESM2_ESM.tif]

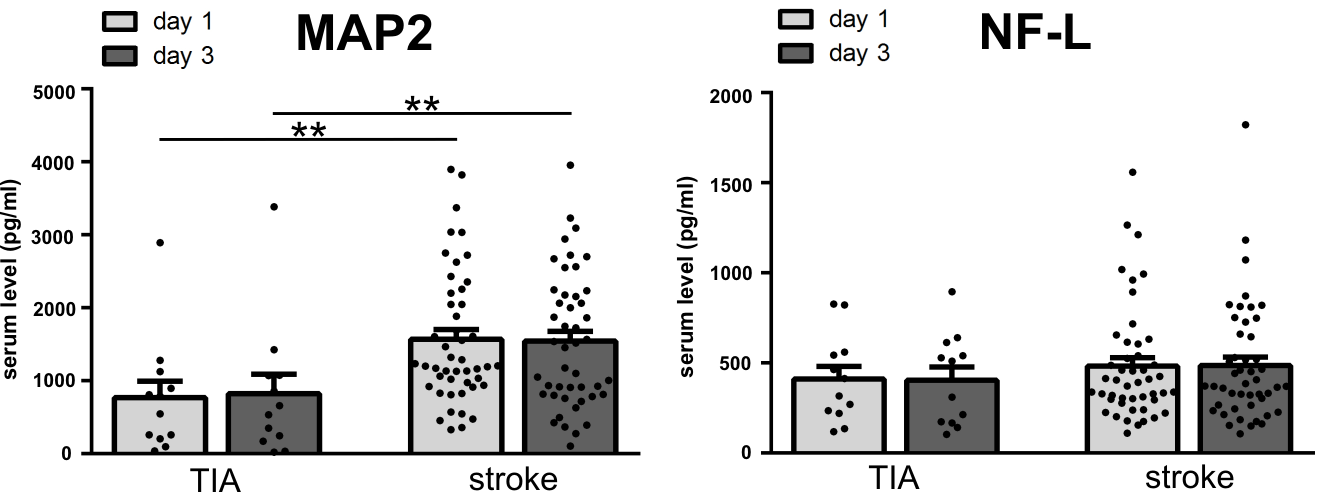

Supplement: Supplementary file 5 — MAP2 and NF-L serum levels of patients suffering from stroke at day 1 and day 3 after stroke-onset: TIA patients are compared to stroke patients (combined group, includes patients with and without intervention). Serum samples of stroke patients exhibit significantly higher serum concentrations of MAP2 than patients suffering from TIA. Bars represent mean values; dots represent individual values; error bars indicate the standard error. TIA: n=12, stroke: n=46-47. **p<0.01. (PNG 103 kb) [file 12035_2021_2372_Fig10_ESM.png]

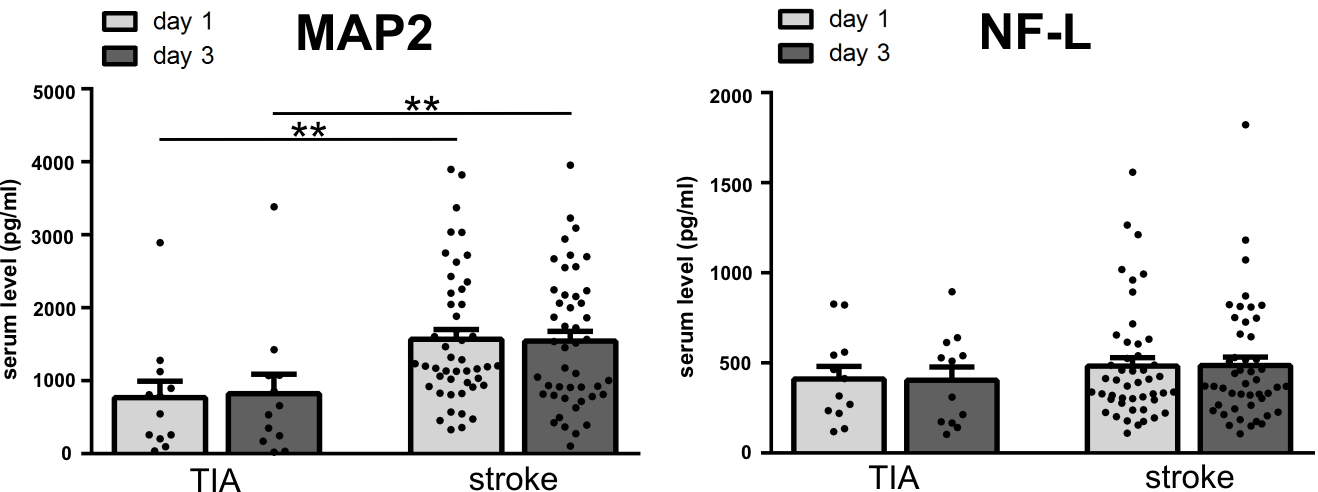

Supplement: Supplementary file 6 — High resolution (TIF 1901 kb) [file 12035_2021_2372_MOESM3_ESM.tif]
